# An extremely rare disconnection of the external iliac artery and novel collateral remodeling in an endometrial stromal sarcoma woman

**DOI:** 10.1186/s12905-022-01746-6

**Published:** 2022-05-11

**Authors:** Shixuan Wang, Ting Zhou, Nan Yu, Ronghua Liu

**Affiliations:** Department of Obstetrics and Gynecology, Tongji Hospital, Tongji Medical College, Huazhong University of Science and Technology, Wuhan, 430030 People’s Republic of China

**Keywords:** External iliac artery, Laparoscopic pelvic lymphadenectomy, Multidisciplinary team

## Abstract

**Background:**

Injury to the external iliac artery can have serious consequences and can be extremely challenging for surgeons. Here, we report a patient with bizarre disconnection of the external iliac artery during a laparoscopic operation.

**Case presentation:**

On May 27, 2020, during a laparoscopic pelvic lymphadenectomy operation to treat endometrial stromal sarcoma, we encountered an unusual anatomy: abnormal disconnection of the left external iliac artery in a 26-year-old female patient. The proximal and distal ends of the left external iliac artery demonstrated old narrowing without active bleeding, and the distance between the two disconnected ends was more than 3 cm. The scenario was surprising to all the staff in the operating theater. After a comprehensive assessment of skin temperature, arterial pulsation and arterial blood flow, a multidisciplinary team determined that collateral circulation of the left lower limb had been established and could meet the blood supply of the lower limbs, which was also confirmed three times by computed tomography angiography and Doppler ultrasound of the blood vessels in the abdomen and lower limbs. Sixteen months after the operation, the patient had no obvious abnormality, and the daily activities of the left lower limb were not affected. Follow-up after treatment for the patient is still in progress.

**Conclusions:**

We describe the details of the whole case of disconnection of the external iliac artery. We hope to summarize the experience and lessons learned through this case and a relevant literature review to improve the safety and orderliness of our future clinical work.

## Background

Common abnormal conditions of the external iliac arteries include congenital malformation, occlusion, traumatic and iatrogenic rupture and even disconnection. The relationship between the pelvic and peritoneal arteries and veins of the human body is not invariable, especially the relationship among the total iliac system and the internal and external iliac arteries and veins, which are very diverse [1, 2]. Such differences include changes in the relative position of or differences in the origins or branches of the blood vessels and a missing or larger trunk; external iliac artery injuries caused by trauma are more common in pelvic fractures and other blunt injuries, which usually start abruptly and quickly lead to hemorrhagic shock [3]. External iliac artery occlusion in these cases is not rare and needs to be treated with bypass grafting if there is no collateral remodeling. The main causes of intimal fibrosis are long-term movement, arteriosclerosis, atherosclerosis, and parasitic diseases, and it tends to occur in bikers and older people [4]; however, some examples have been found in young patients. Arterial endofibrosis is an artery disease that occurs in endurance athletes and may result in excessive iliac artery length and blood flow limitations [5]. Intimal fibrosis of arterial endofibrosis can, in turn, lead to external iliac artery occlusion due to infection, tumor thrombus and surgery. The major causes of iatrogenic external iliac artery injury are puncture, electric burning, suturing and excessive pulling. The scope of gynecological surgery is concentrated in the pelvic cavity. The external iliac artery is an important large vessel located in the pelvic lateral wall vessel area that is prone to injury. In addition, the incidence of thrombosis after gynecological surgery is also high.

Here, we report a patient with a bizarre disconnection of the external iliac artery, but far too many questions remain that could not be resolved. We describe the details of the whole case in the following text. We hope to summarize the experience and lessons learned through this case and a relevant literature review to improve the safety and orderliness of our future clinical work.

## Case presentation

**Fig. 1:**
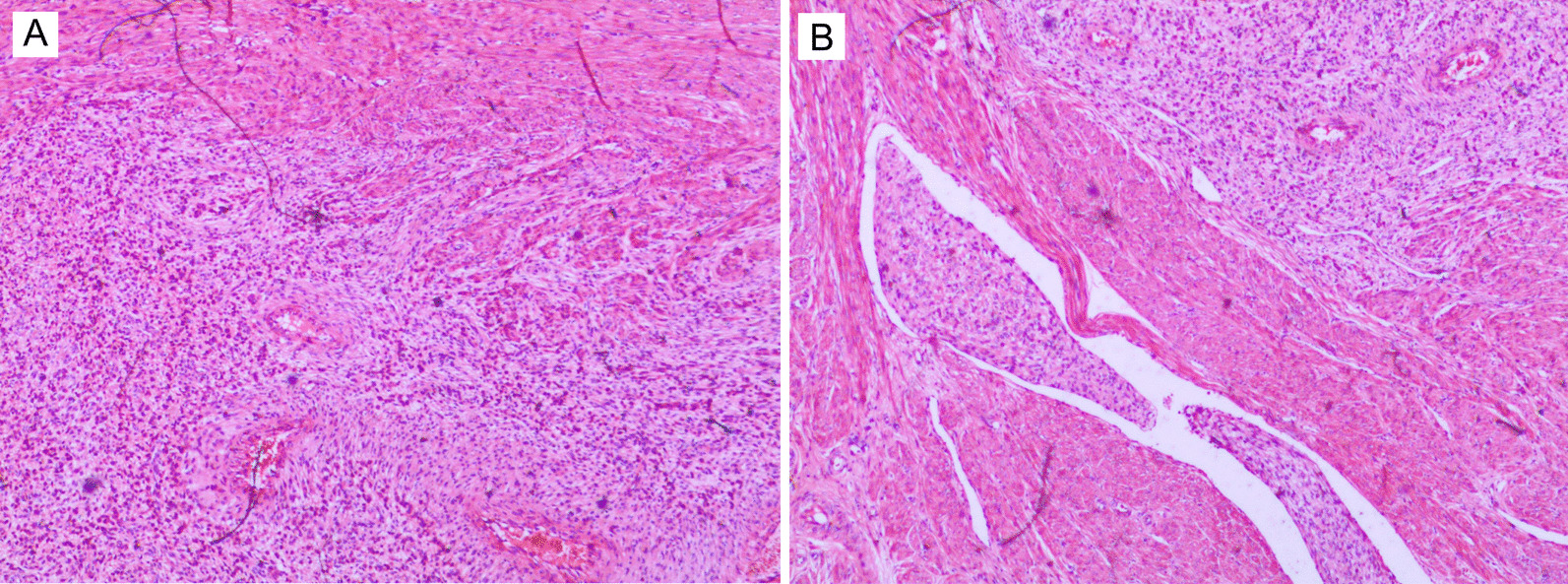
Pathological results of the two operations. A low-grade endometrial stromal sarcoma and a tumor thrombus in the vessels are shown in **A** and **B** at the first operation. No tumor metastasis was found in any lymph node (**C**, **D**)

The unmarried female patient was 26 years old with normal menstruation. At the age of 13 years, she experienced menarche. The last menstruation started on April 27, 2020 and lasted approximately ten days. She denied a history of congenital or acquired bleeding disorders, liver disease, or use of antiplatelet drugs, anticoagulants, or nonsteroidal anti-inflammatory drugs. The patient indicated that she started smoking when she was 16 years old. In recent years, she has smoked more than 20 cigarettes a day. Furthermore, she had been working in a nightclub and drank every day. In addition, the patient had a bad fall and landed on the ground in the bath. Because of this abnormal menstruation, she went to a local hospital, and color Doppler ultrasound showed 8 × 9 × 7 cm^3^ uterine fibroids. On May 6, 2020, the patient underwent abdominal myomectomy in that hospital. During the operation, a myomatous protrusion (7 × 6 × 5 cm^3^) was found in the posterior wall of the uterus. The muscle layer of the surface was cut, revealing a brown mass with an uneven surface and no obvious capsule. The mass was a fusion of several small masses that had penetrated the endometrial layer. The pathological results after the operation showed low-grade endometrial stromal sarcoma with hemorrhagic necrotic cystic changes and a tumor thrombus in the tumor vessels (Fig. 1A, B).

The patient returned to our hospital 10 days after the operation. Until this point, she had no complaints of obvious discomfort, a normal gait, no obstacles to lower limb movement and no history of left leg pain associated with walking and relieving by rest. There was no significant difference in muscle tension or skin temperature between the two legs and no claudication in her left leg. The results of serum tumor markers showed a CA125 level of 68.8 U/ml (< 35.0 U/ml), and normal levels of alpha-feroprotein (AFP), carcinoembryonic antigen (CEA), CA15-3, CA19-9, human epididymis protein 4 (HE4) and squamous cell carcinoma antigen (SCCA). After admission, coagulation function showed that the D-dimer level was 1.86 µg/ml, the activated partial thromboplastin time was 43.9 s (29.0–42.0 s), and other indexes were normal. Ultrasound showed that the volume of the uterus was 5.5 × 4.5 × 6 cm^3^, and the echo of the posterior wall of the uterus was disordered. Pelvic magnetic resonance imaging (MRI) evaluation showed that the endometrium was slightly thickened and that the signal of the myometrium was uneven on May 18. Diffusion-weighted imaging (DWI) showed no obvious, abnormally high signal or abnormally enhanced focus in either the pelvic floor muscle or pelvic bone. Pelvic MRI/DWI imaging before laparoscopic surgery could not reflect abnormalities of the external iliac artery.

**Fig. 2:**
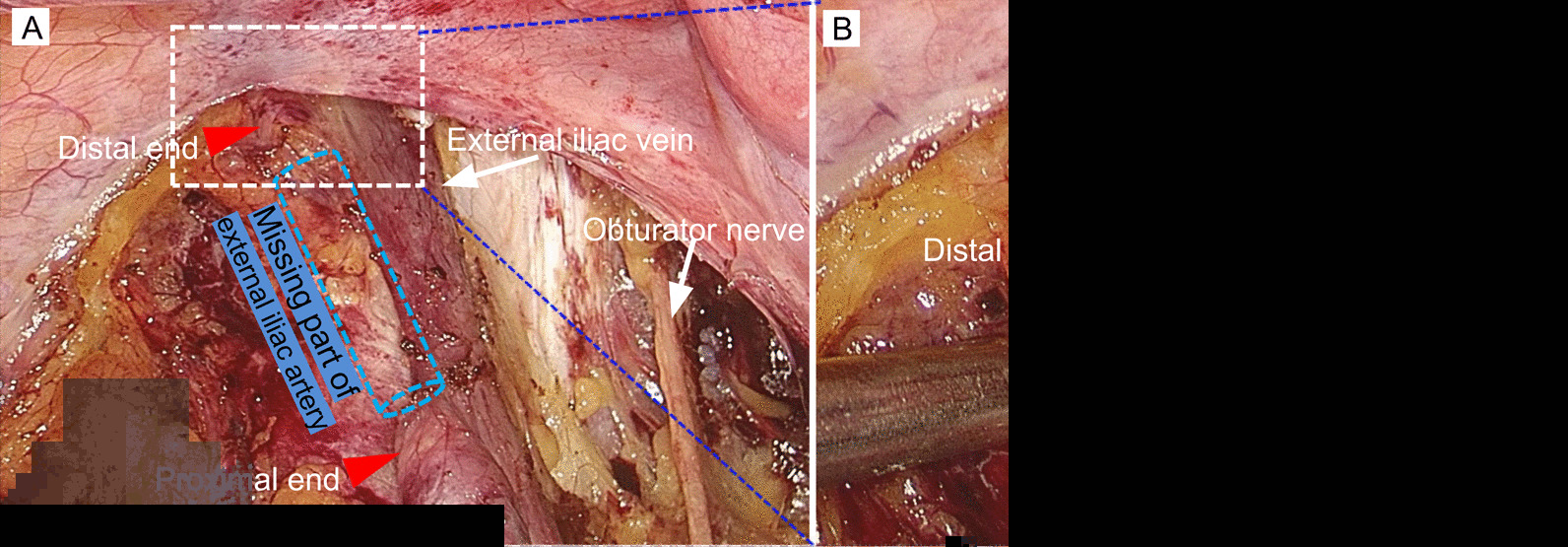
Abnormal left external iliac artery found during laparoscopic surgery. The severed left external iliac artery is shown in **A**, and the distal end of the left external iliac artery is shown in **B**. Red arrowheads indicate the two ends of the artery. The dotted column indicates the distance between the two ends

The laparoscopic operation showed that the sigmoid colon, a portion of the rectum and the left side of the pelvic wall were densely adhered, and the uterus and double appendages were difficult to expose. After lymphadenectomy of the paraaortic and right pelvic lymph nodes, the left peritoneum was opened to expose the left iliac vessels during resection of the left pelvic lymph node. Approximately 2.5 cm from the bifurcation of the left common iliac artery, the left external iliac artery was found to be discontinuous (Fig. 2). The diameter of the left external iliac artery was significantly smaller than that of the right side. There were no electrocautery changes, acute inflammation or perivascular hemorrhage preferentially surrounding the left external iliac artery.

**Fig. 3:**
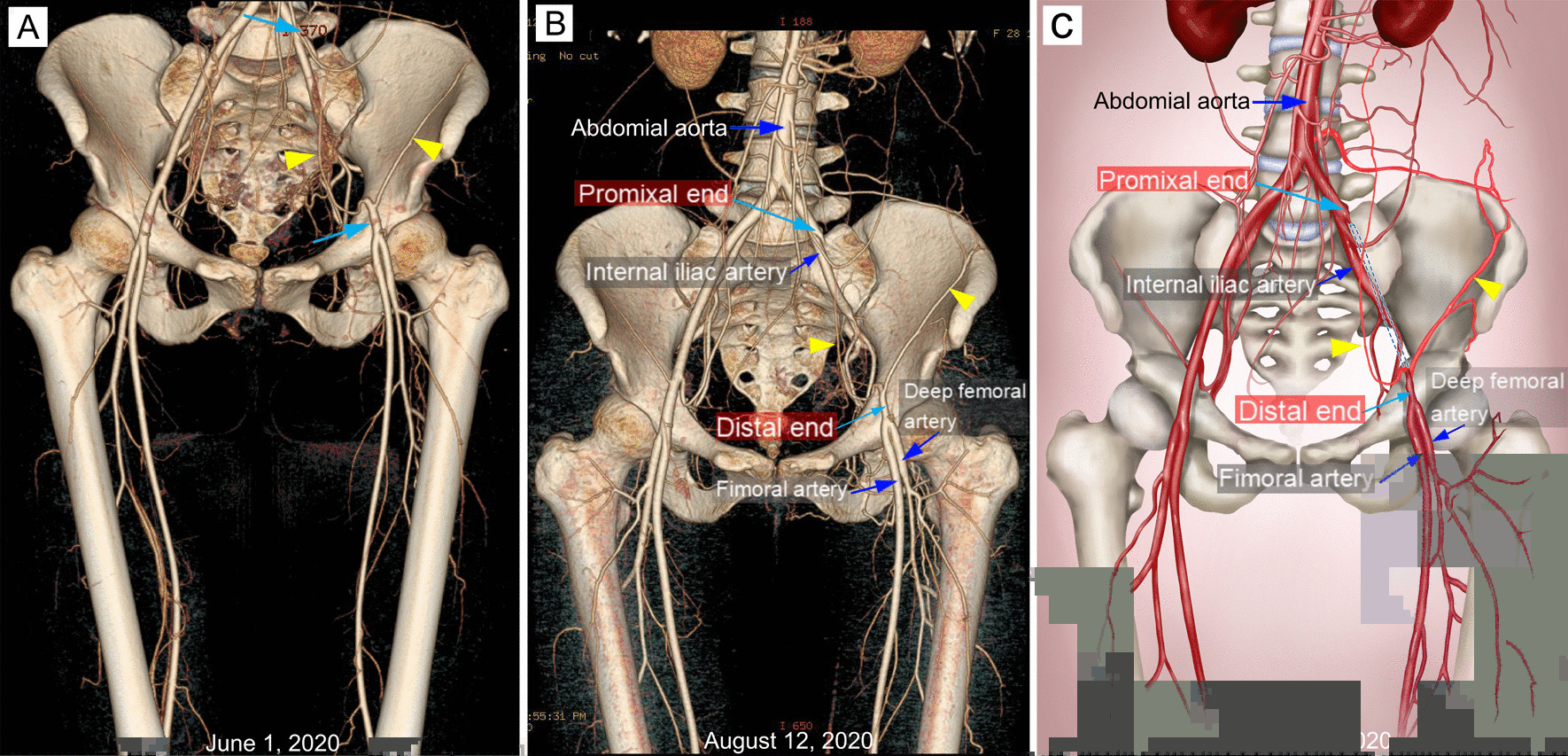
Imaging of the left external iliac artery and other vessels of the bilateral lower limbs with computed tomography angiography (**A** on the sixth day after the second operation; **B** three months after operation) and correlative illustration of **B** (**C**). Collateral circulation was visible in the left external iliac artery (yellow arrows), indicating that the blood supply of the left lower limb was not affected

A surgical multidisciplinary team (MDT) consultation was launched, and the severity of the emergency was evaluated. Arterial pulsation in the left dorsalis pedis artery was weaker than that in the right artery. The skin temperature and color of the left foot were normal, and there was no significant difference in the body temperatures of the two lower extremities. She had palpable left dorsalis pedis and posterior tibialis pulses. The blood flow spectrum of the femoral artery could be seen by color Doppler ultrasound during the operation. After a comprehensive assessment of skin temperature, arterial pulsation and arterial blood flow, the experts suggested that the status of the disconnection of the left EIA was nonacute and indicated that an organic thrombus may have been present in the lumen of the artery. In addition, collateral circulation of the left lower limb was established and could meet the blood supply of the lower limbs, which was also confirmed by computed tomography angiography (CTA) on June 1, 2020. Collateral circulation was visible in the left external iliac artery (Fig. 3A). Daily activities such as walking were not affected before the second operation. The experts agreed that there was no need to use artery bypass to establish a new collateral circulation. Because the two blind ends of the left external iliac artery were far away from each other, it was impossible to perform end-to-end anastomosis; therefore, the blind ends were ligated with 10 silk threads.

**Fig. 4:**
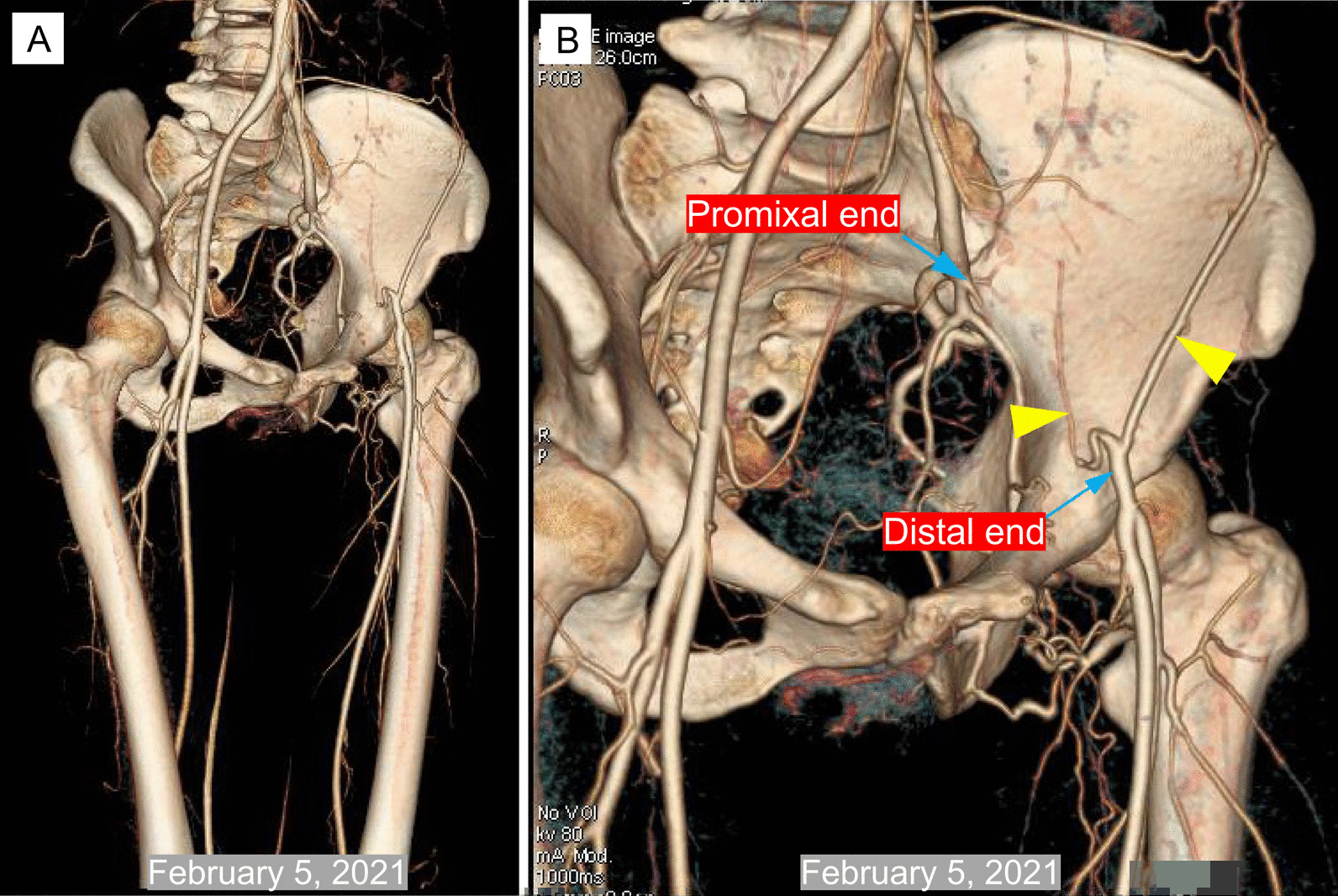
Computed tomography angiography imaging of the bilateral lower limbs on February 5, 2021 (**A**, CTA image of lower limb vessels; **B**, abnormal left external iliac artery and collateral circulation in the left external iliac artery)

**Fig. 5:**
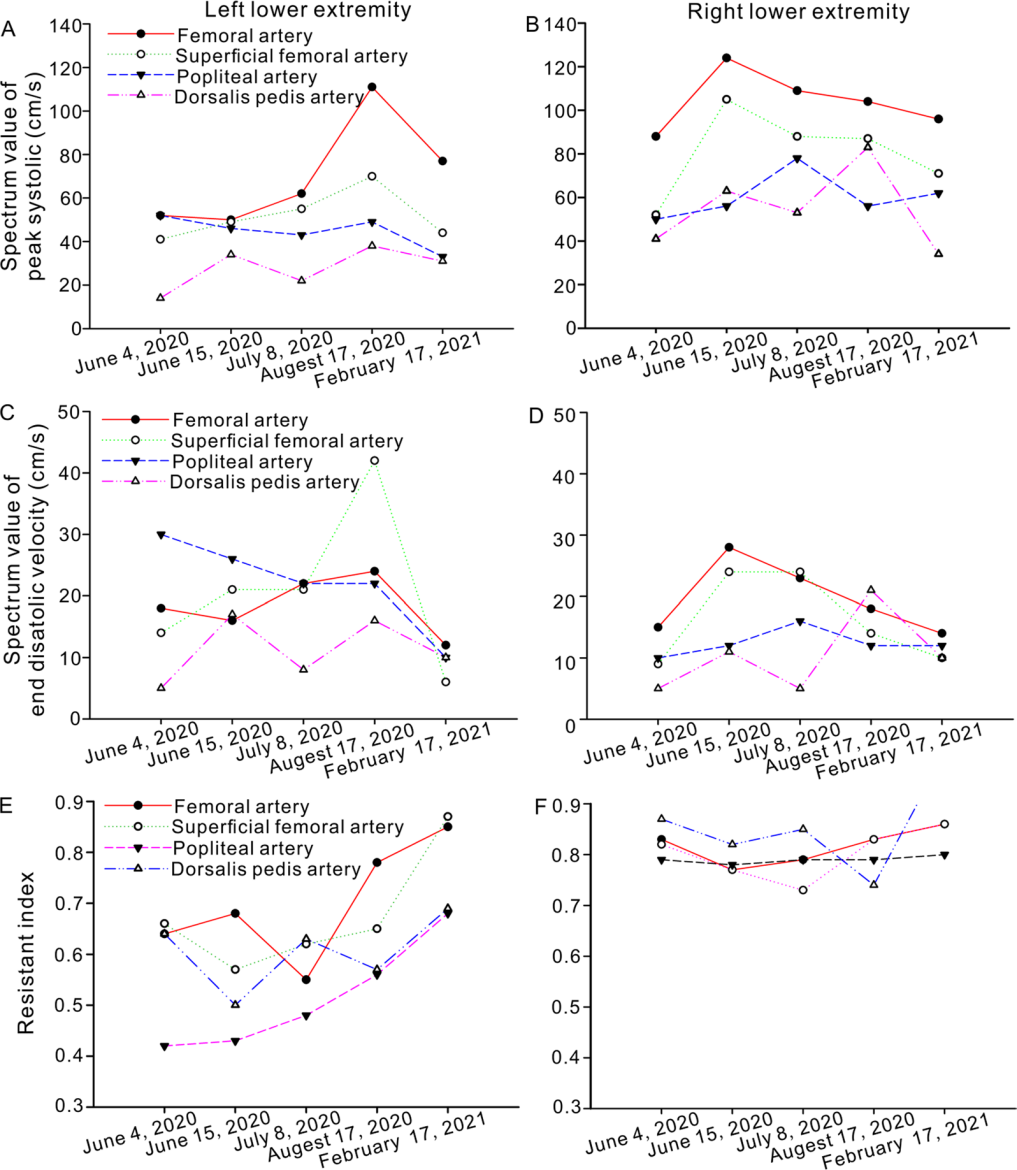
Arterial blood flow of the lower extremities after the second operation. The peak systolic and end diastolic blood flow velocities and the resistance indexes of the bilateral lower extremity arteries are shown in **A**–**F** at different times after the second surgery

**Fig. 6:**
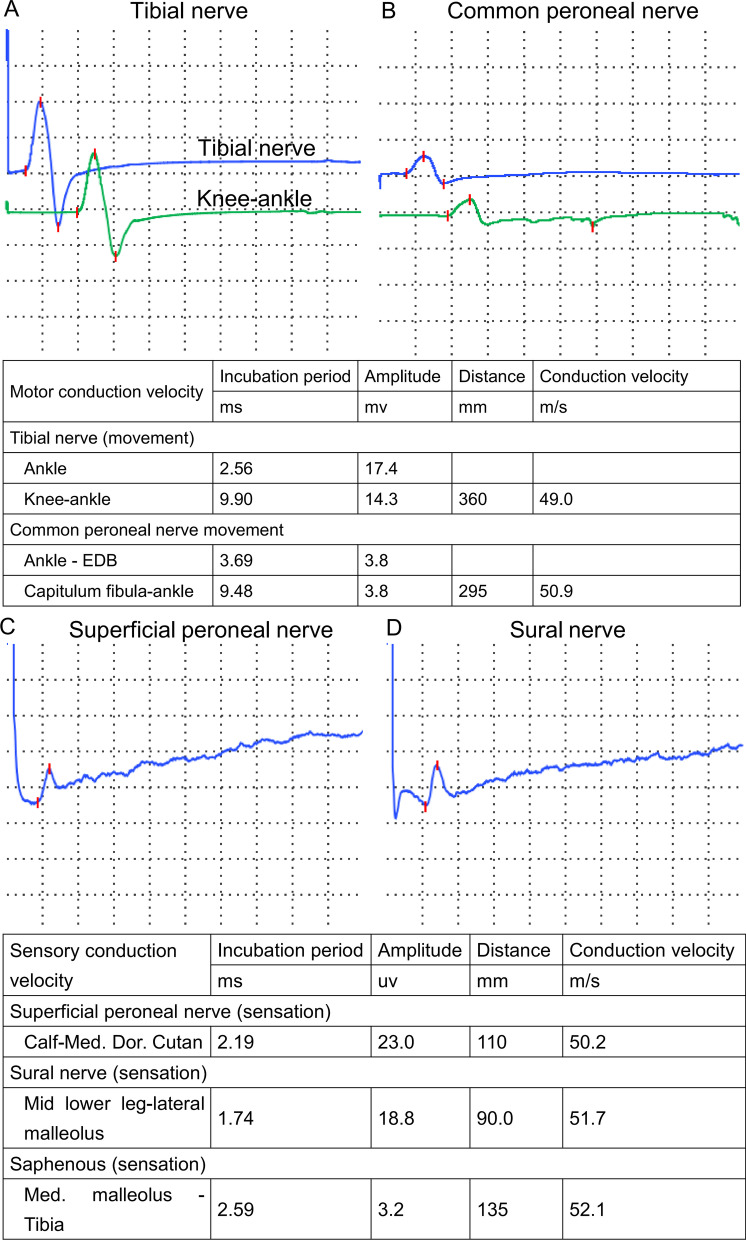
Nerve examination of the left lower extremity. The compound muscle action potential (CMAP) of the tibial nerve and peroneal nerve of the left lower limb is shown in **A**, **B**, and the sensory nerve action potential (SNAP) of the superficial peroneal nerve and sural nerve of the left lower limb is shown in Panels **C**, **D**

**Fig. 7:**
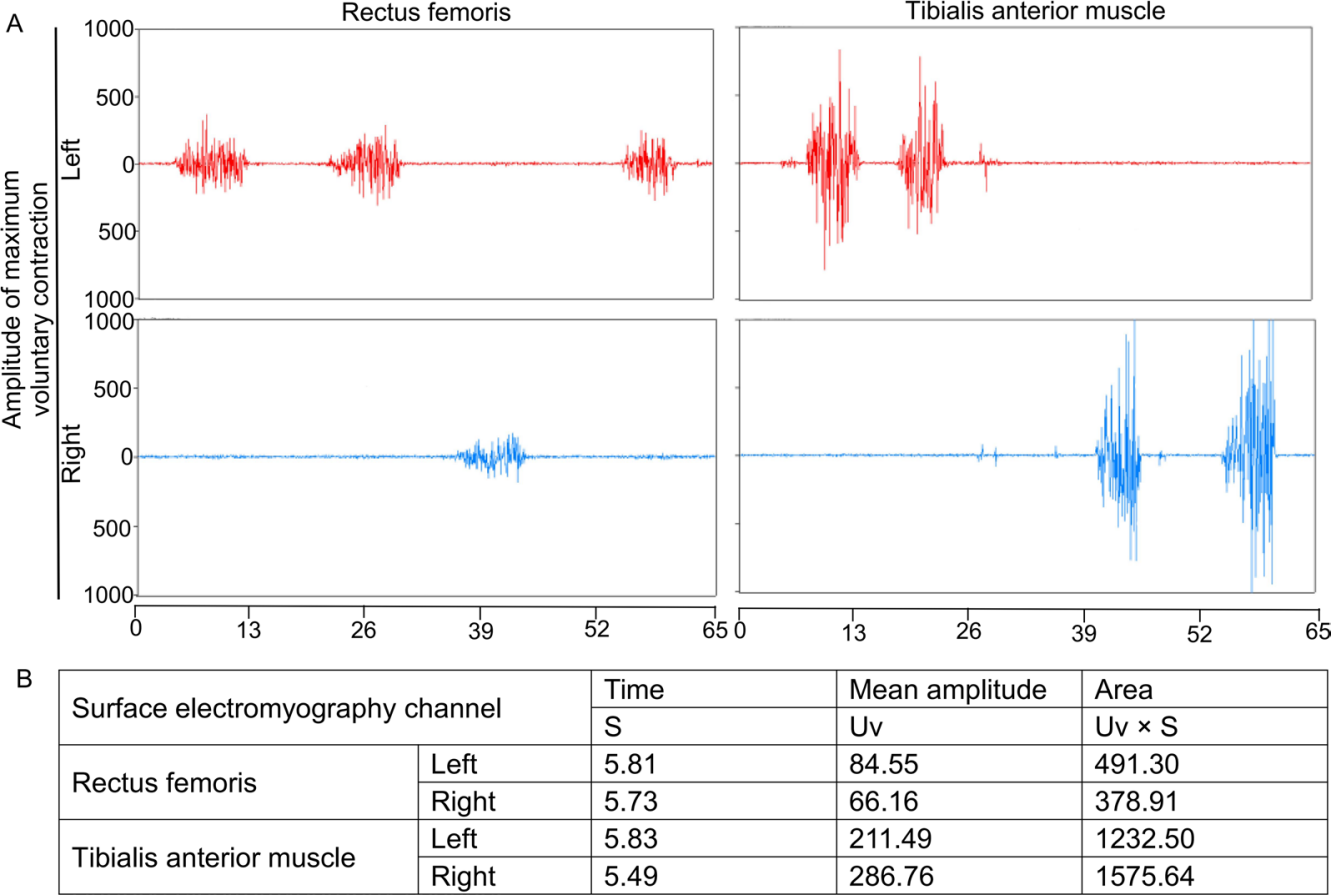
Muscle examination of the lower limb. The maximum voluntary contraction (MVC) of the bilateral rectus femoris and anterior tibialis muscles is shown in **A**, **B**

After the second operation, another two CTA results (Augest 12, 2020 and Feburary 5, 2021) after the operation showed that no new branch was involved in the collateral circulation network at the proximal broken end (Figs. 3B, C and 4). Ultrasound was used to evaluate the blood flow of the main arteries of the lower limbs four times after the second operation. Compared with that of the arterial blood flow of the opposite lower extremity, the spectrum value of the peak velocity of the femoral artery, superficial femoral artery, popliteal artery and dorsalis pedis artery in the left lower limb was not greatly affected by the presence of the abnormal left external iliac artery (Fig. 5A–D). Furthermore, the resistance index of the four main arteries of the left lower limb was lower than that of the arteries of the contralateral lower limb (Fig. 5E, F). At the same time, electromyography was used to analyze the effect of the abnormal external iliac artery of the left lower extremity. No obvious abnormality was found in the compound muscle action potential (CMAP) of the tibial nerve or peroneal nerve or in the sensory nerve action potential (SNAP) of the superficial peroneal nerve or sural nerve of the left lower limb (Fig. 6). There was no significant difference between the bilateral lower limbs in the maximum voluntary contraction (MVC) of the bilateral rectus femoris and anterior tibialis muscles (Fig. 7).

**Fig. 8:**
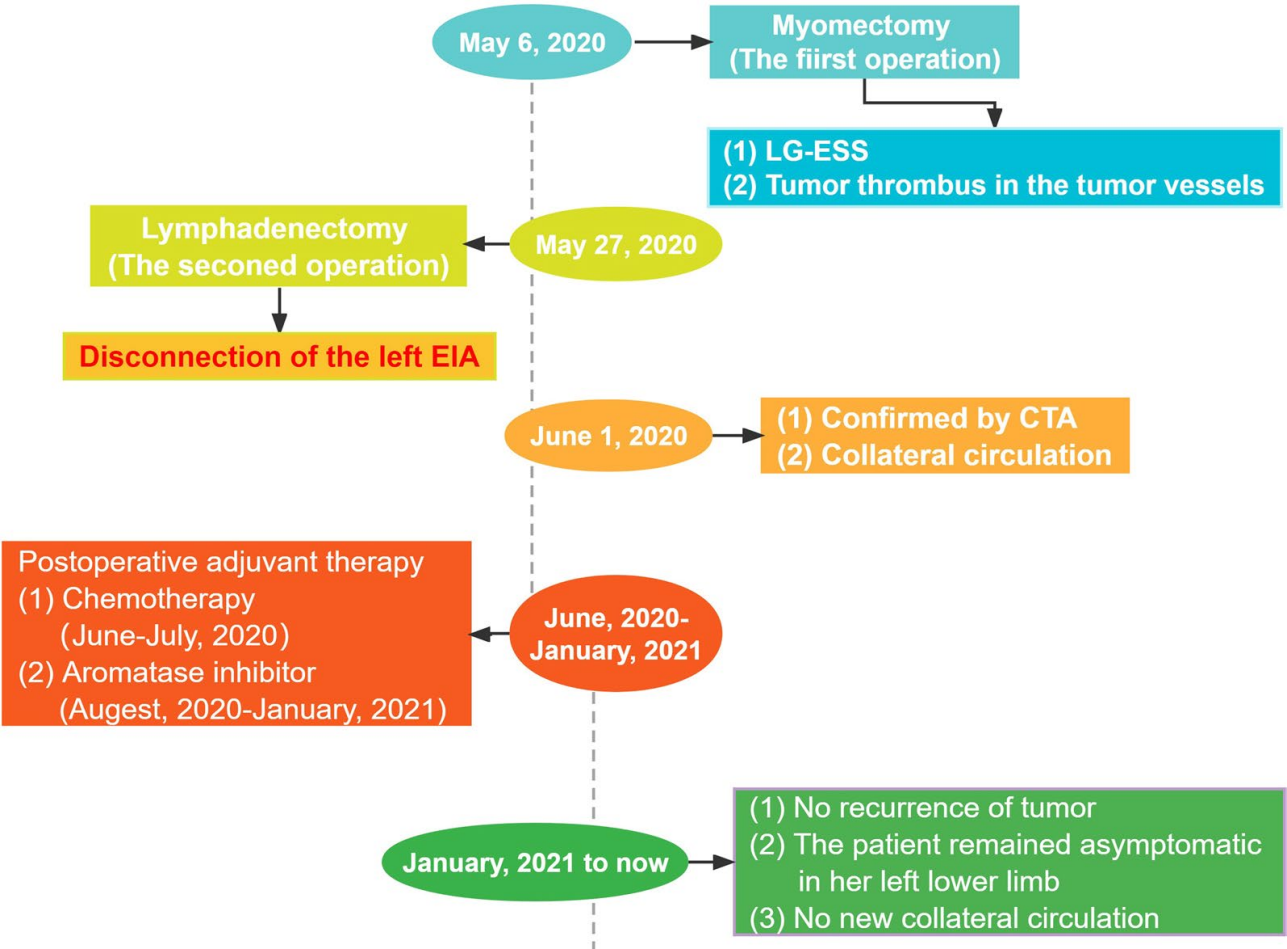
The timeline of surgery/imaging and follow-up of this case

The patient was diagnosed with stage IB endometrial stromal sarcoma. As the patient underwent fertility-sparing surgery, a tumor thrombus was observed in the tumor vessels. Therefore, after fully communicating with the patient and signing the informed consent form, she received liposomal doxorubicin intravenous chemotherapy (30 mg/m^2^) and oral aromatase inhibitors (letrozole, 2.5 mg once daily) for six months. We managed this particular case as threatening but not dangerous. After surgery, pelvic MRI revealed that the uterus showed postoperative changes, and no abnormal signal was found in the uterus sixteen months after the operation. There was no obvious abnormality in her left lower limb, no claudication had developed, and the patient remained asymptomatic. The timeline of surgery/imaging and follow-up of this case is shown in Fig. 8.

## Discussion and conclusions

As an important main artery of the lower limbs, the external iliac artery is one of the final branches of the common iliac artery. Common abnormal conditions of the external iliac arteries include congenital malformation, occlusion, traumatic and iatrogenic rupture and even disconnection. Injury to the external iliac artery can have serious consequences, such as fatal intraabdominal hemorrhage, lower extremity amputation and even life-threatening conditions, all of which can be extremely challenging for surgeons [6–8]. Artery reconstruction can be treated with prosthetic patches or by grafting with end-to-end anastomosis [9]. Even if patients survive to undergo extra-anatomic bypass, the major complication rates may exceed 40% [10]. The mortality rate among patients undergoing damage to the common or external iliac artery ranges from 30 to 90% and is often a direct result of massive blood loss [11]. The major causes of iatrogenic external iliac artery injury are puncture, electric burning, suturing and excessive pulling. Overall, the incidence of vascular injuries is rare, estimated to be between 0.9 and 2.3 per 100,000 individuals [12]. If this emergency occurs, doctors should perform emergency treatment correctly and quickly and rely on an MDT team to properly discuss the necessary procedures.

To our knowledge, no similar reports have been published in the literature. We reflect on what caused the situation and how to address it. There were serious, dense adhesions in the pelvic cavity, and the left external iliac artery was found to be discontinuous during resection of the left pelvic lymph node. Bizarrely, the left external iliac artery had no active bleeding at all, and the proximal and distal ends showed old constriction and blind end changes. Combined with the above analysis, the possibility of congenital disconnection of blood vessels is very small. No lacerations were observed, and there were no ischemic symptoms or changes in the left lower limb, except for a slightly weaker pulse of the left dorsal foot than of the right dorsal foot. How, then, did this important artery become disconnected?

We are unsure whether chronic disease or acute vascular injury had occurred during the first operation. Latrogenic vascular injury is a potentially serious complication of many surgical procedures. Is this vascular anomaly congenital or compensatory collateral circulation after EIA injury? Injury to the EIA during the first operation would have been serious; however, it did not occur during that operation. On the other hand, the scope of myomectomy is not in the location of EIA. Whether it was caused by her own abnormal physical condition. External iliac artery endofibrosis (EIAE) can cause arterial thrombosis and rupture [13–17]. EIAE is defined as a specific intimal thickening of the wall of the artery. The precise pathophysiologic mechanism of EIAE is unclear [18]. EIAE is a rare disease seen primarily in young healthy endurance athletes. Athletic cyclists easily formed external iliac artery endofibrosis and thrombosis. Anatomical, mechanical, and metabolic factors may be the causes of EIA [19]. The patient’s smoking for more than 10 years (more than 20 cigarettes every day), excessive drinking, working in a nightclub and having a history of sitting on the ground after wrestling may have predisposed her to EIAE. Moreover, the long-term severe pelvic adhesion observed in this operation may aggravate EIAE. These elements may be the causes of arterial disease.

The CT images showed that the collateral circulation originated from the distal end of the EIA, which participated in the collateral circulation network between the two ends. From the results of four bilateral lower extremity vascular ultrasound examinations, it can be seen that the resistance index of the four main arteries of the left lower limb was lower than that of the opposite side. Is this a coincidence, or was this a new collateral circulation that formed after the EIA had been severed? We are unable to clearly judge the situation at this time. In the existing literature, we found no similar abnormal collateral circulation of EIA. This may be the first time that this variation in the external iliac artery has been discovered.

The patient’s mother claimed that there were no obvious abnormal changes in either lower limb until after the first operation, but after the second operation, the situation changed. The patient continued to feel weakness of the left lower limb if she walked for a long time. Three months after the second operation, the symptoms gradually disappeared. This meant that the abnormal symptoms in the left EIA may have been related to surgery. Managing these patients after treatment is extremely confusing and challenging. Whether the current collateral circulation can compensate for major vessel occlusion, maintain a well-perfused foot and meet the needs of daily life is unknown and remains to be investigated. We will closely observe the changes in the blood vessels of the patient’s lower limbs and their impact on her daily activities and take corresponding treatment measures according to the situation at hand.

In conclusion, the abnormal condition of the left external iliac artery remains to be fully understood. The time of origin also remains unknown. Based on the treatment administered to this patient, we deem that as long as no acute blood loss occurs, even after such a serious arterial injury, it is possible to reestablish the collateral circulation to meet the needs of the limbs. Of course, this collateral circulation may have developed on its own. In the process of surgery, if a similar situation occurs, it would not be necessary to rush to manage the severed blood vessels. The MDT must conduct a comprehensive assessment of the blood vessels, muscles, limb movement and neurological function as quickly as possible before treatment. It is hoped that this case can provide a reference for clinicians to address similar critical situations.

## Data Availability

The datasets used during the current study available from the corresponding author on reasonable request.

## Abbreviations

EIA: External iliac artery
MDT: Multidisciplinary team
CTA: Computed tomography angiography
EIAE: External iliac artery endofibrosis
CMAP: Compound muscle action potential
SNAP: Sensory nerve action potential
MVC: Maximum voluntary contraction
